# Direct and Indirect Effects of Resource P-Limitation Differentially Impact Population Growth, Life History and Body Elemental Composition of a Zooplankton Consumer

**DOI:** 10.3389/fmicb.2018.00172

**Published:** 2018-02-09

**Authors:** Libin Zhou, Kimberley D. Lemmen, Wei Zhang, Steven A. J. Declerck

**Affiliations:** ^1^Department of Aquatic Ecology, Netherlands Institute of Ecology (NIOO-KNAW), Wageningen, Netherlands; ^2^Jiangxi Provincial Key Laboratory of Water Resources and Environment of Poyang Lake, Jiangxi Institute of Water Sciences, Nanchang, China

**Keywords:** phosphorus limitation, *B. calyciflorus*, population growth, life history, organismal stoichiometry

## Abstract

One of the central tenets of ecological stoichiometry is that consumer growth rate is strongly determined by food phosphorus (P) content. In planktonic organisms population growth rates of zooplankton have repeatedly been shown to be reduced when fed with P-limited algal food sources. However, P-limitation may also affect other quality-related aspects of algae, such as biochemical composition or palatability. We studied the population growth, detailed life history and body elemental composition of the herbivorous rotifer, *Brachionus calyciflorus*, in response to three different food quality treatments: algae cultured in high phosphorus conditions (average algal molar C:P ≈ 112, ‘HP’), algae cultured in low P conditions (molar C:P ≈ 631, ‘LP’) and low-P cultured algae spiked with P just before feeding (molar C:P ≈ 113, ‘LP+P’). LP+P algae thus combined high P content with a history of growth under P-limited conditions. Total P content and the C:P ratio of rotifers in the LP+P treatment equaled those of rotifers in the HP treatment. Rotifer population growth rates were higher in HP than in LP and intermediate in the LP+P treatment. Similarly, many life history traits observed for animals in the LP+P treatment, such as somatic growth rate, age at maturity, and egg production rate were also intermediate to those observed in the LP and HP treatments. However, there were important deviations from this pattern: size at first reproduction and egg mortality in the LP+P treatment equaled the HP treatment, whereas size and development time of the first eggs equaled those of the LP treatment. Our results indicate that elemental limitation cannot fully explain reduced performance of consumers fed with P-limited algae and strongly suggest that indirect, non-stoichiometric effects of P-limitation, e.g., via changes in biochemical composition or morphology of the algae also play a major role. Furthermore, our study highlights that such indirect effects have a differential impact on major fitness components and may as such also determine the population dynamics and demographic structure of consumer populations.

## Introduction

As a major component of the macromolecules DNA, RNA, and ATP, phosphorus (P) is an essential element for the growth and reproduction of organisms. Due to this dependence, the availability of P may strongly limit the productivity of primary producers and higher trophic levels (Hessen, 1992; DeMott and Gulati, 1999; McCarthy et al., 2006). Human activities increasingly alter the amounts and ratios of biogenic elements (e.g., carbon, nitrogen, and phosphorus) in natural systems and cause many freshwater systems to become P-limited (Stockner et al., 2000; Elser et al., 2009). A better mechanistic understanding of how P-limitation impacts the organisms in these ecosystems is therefore urgently needed.

Laboratory studies have shown strong reductions in the growth and reproduction of primary consumers when fed even high amounts of P-limited food (Sterner and Hessen, 1994; Sterner and Schulz, 1998). Such reduced performance has stimulated considerable debate about the underlying mechanisms. One potentially important cause of reduced consumer performance is pure mineral limitation: when the food resource has a very low P-content, the supply to a consumer may be too low even when food intake of the latter is at its maximum (Sterner and Hessen, 1994; DeMott, 1998). Furthermore, stoichiometric mismatches between the nutrient content of producers and consumers may also incur costs for the consumer, such as those associated with the disposal of excess C and other elements (Darchambeau et al., 2003). However, in addition to such direct effects, P-limitation may also affect the quality of producers indirectly. P-limitation in algae, for example, has been shown to decrease the amount of highly unsaturated fatty acids (Müller-Navarra, 1995; Weers and Gulati, 1997b; Spijkerman and Wacker, 2011; Challagulla et al., 2015) which are important components for consumer growth and reproduction (Weers and Gulati, 1997a; Ravet and Brett, 2006). P-limitation has also been shown to result in changes of algal cell size and cell wall morphology (van Donk and Hessen, 1995; van Donk et al., 1997). Lürling and van Donk (1997) and van Donk et al. (1997) explained reduced performance of *Daphnia* grown on P-limited algae by the lower digestibility of their thickened cell walls. DeMott (1998) demonstrated that the performance of *Daphnia* may be limited by energy even when fed high C:P algal food because of the low digestibility of P-deficient algae. These studies thus all indicate that food P-limitation may negatively affect consumers in direct as well as indirect, non-stoichiometric ways.

Ecological stoichiometry (Sterner and Elser, 2002; Hessen et al., 2013) has so far been the predominant framework contributing to a better understanding of the impact of nutrient limitation and stoichiometric mismatch on primary and secondary productivity (Malzahn et al., 2010), grazer top down control and nutrient cycling (Sistla and Schimel, 2012), the strength of trophic cascades (Hall, 2009) and trophic transfer efficiency (Rowland et al., 2015). Potentially, stoichiometric models still underestimate the full impact of nutrient limitation because indirect effects are typically not taken into account. The general lack of consideration of such indirect effects probably results from our poor understanding of the causal mechanisms underlying such effects, from the scarcity of information on their relative importance and from the difficulties inherent to incorporating these effects in mathematical models.

**FIGURE 1:**
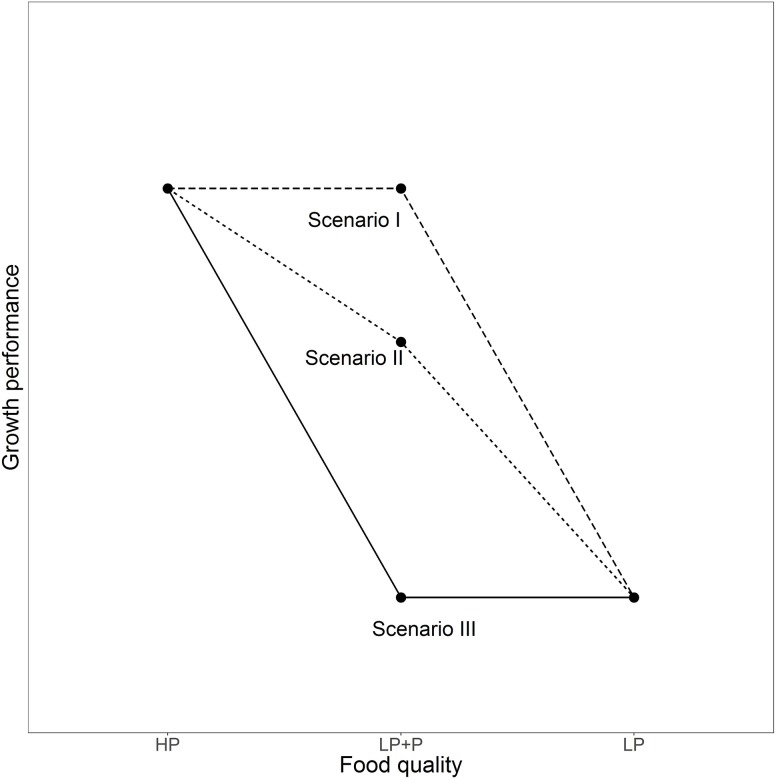
Three potential scenarios of how the performance of consumers may respond to the food quality treatments in a P-supplementation experiment. Scenario I depicts a case where the growth reduction of consumers fed P-depleted food is uniquely caused by direct, stoichiometric effects of P-limitation. Conversely, in Scenario III, this reduction in growth performance is entirely due to non-stoichiometric indirect effects of P-limitation. In scenario II both direct and indirect effects are of large importance. HP: P-saturated food; LP: P-deficient food; LP+P: P-deficient food enriched with a P-supplement.

P-supplementation tests may provide us with a powerful experimental tool to address the relative importance of indirect, non-stoichiometric effects, even when knowledge about the causes is lacking. The approach makes use of the fact that P-limited algae are able to quickly absorb inorganic P from their environment (Lehman and Sandgren, 1982) and hinges on the assumption that the process of P-uptake is much faster than responses in other traits, such as abundance, biochemical composition or morphological features (Boersma, 2000; Elser et al., 2001). The relative importance of direct stoichiometric and indirect non-stoichiometric effects can be estimated through a comparison of the performance of consumers fed equal biomasses of P-replete (HP), P-limited (LP), and P-supplemented LP algae (LP+P). Equal performance of consumers in the LP+P as in the HP treatment indicates that direct P-limitation is the only cause of reduced performance in the LP treatment (**Figure 1**, Scenario I). Conversely, low consumer performance in the LP treatment can completely be attributed to indirect effects of P-limitation if P-supplementation results in no improved consumer performance compared to the LP treatment (**Figure 1**, Scenario III). If performance of consumers in the LP+P treatment is intermediate to the LP and HP treatments, then the relative importance of direct and indirect mechanisms can be inferred from the position of the LP+P treatment compared to LP and HP (**Figure 1**, Scenario II). A key requirement is that algae in the LP+P treatment acquire a C:P ratio equal to the HP algae.

Only few studies have used such experimental approach to evaluate the relative importance of direct and indirect effects of P-limitation on consumers. Rothhaupt (1995) found that although supplementation of P-limited algae enhanced the exponential population growth rate of the rotifer *Brachionus rubens*, it still remained considerably below that in P-rich algae and he suggested biochemical limitation as the mechanism underlying the observed indirect effect. DeMott (1998) found strong improvements of somatic growth to P-supplementation of P-limited algae in multiple *Daphnia* species; although growth of most species almost approximated the levels observed with P-rich algae, they still remained somewhat lower in most cases. Boersma (2000) and Becker and Boersma (2003) cross-combined P-treatments (LP, HP, and LP+P) with fatty acid supplementation treatments and concluded that biochemical limitation by fatty acids only becomes important when phosphorus is present in ample supply, and suggested that other factors were still at work since the joint effects of P and highly unsaturated fatty acids could not fully explain the higher growth rate observed in HP algae. Ravet and Brett (2006) demonstrated a stronger negative impact of indirect than direct P-limitation effects on *Daphnia* somatic growth and reproduction.

Nutritional requirements of a consumer organism differ between its life stages. This has been shown for stoichiometric (Urabe and Sterner, 2001; Villar-Argaiz et al., 2002; Færøvig and Hessen, 2003) as well as for biochemical requirements (Martin-Creuzburg and Von Elert, 2004; Boëchat and Adrian, 2006; Wacker and Martin-Creuzburg, 2007). So far, P supplementation studies have mainly assessed the response of consumers to food quality treatments by considering general performance criteria, such as somatic growth (Boersma, 2000; Elser et al., 2001) or population growth (Rothhaupt, 1995). As a result, it remains unclear how the relative impacts of direct and indirect food quality effects vary among life history traits or major fitness components. Such information is, nevertheless, key to a better understanding of the consequences of nutrient limitation on the dynamics and demographic structure of consumer populations.

An implicit assumption of the P-supplementation method is that the accessibility of P to consumers should be equal in both LP+P and HP treatments. This may not necessarily be so. For example, a reduced digestibility of algae associated with P-limitation (van Donk et al., 1997) may result in a reduced availability of P to the consumers. Furthermore, when supplied to P-starved algal cells, anorganic phosphates may initially be stored under the form of polyphosphates in attendance of further metabolization (Eixler et al., 2006). If consumers are less able to take up and assimilate P from polyphosphates than from other P-containing biomolecules (e.g., DNA, RNA, ATP, phospholipids) then polyphosphate storage in LP+P algae could result in a reduced growth of consumers compared to those fed with HP food. To our knowledge, none of the P-supplementation studies so far have considered the possibility that a reduced accessibility of P in LP+P algae to consumers may unduly emphasize the importance of indirect effects.

With this study, using a P-supplementation approach we aimed at studying the relative importance of direct and indirect effects of P-limitation on population growth performance and a variety of life history traits, using the rotifer *B. calyciflorus* as consumer model. In an effort to evaluate whether differences exist in accessibility of P to consumers between LP+P and HP algae, we simultaneously studied the effect of food quality treatments on consumer elemental content and composition. Our results show that, whereas P-supplementation of P-limited algae enhanced P-content of algae as well as of rotifers to levels equal to those of P-replete conditions, population growth, somatic growth as well as individual fitness remained lower, indicating an important impact of non-stoichiometric, indirect effects. These effects seemed to have a differential impact on fitness components as life history traits responded in various ways to the supplementation treatment.

## Materials and Methods

### Rotifer and Algae Cultures

Three clones of the rotifer *B. calyciflorus* were obtained from the resting egg banks of two Dutch lakes (D12 and D61 52°01′31.2″N, 4°11′16.8″E; E1 52°38′41.9″N, 4°43′81.7″E). *B. calyciflorus* consists of a species complex containing at least four putative species (Papakostas et al., 2016). Based on ITS1-sequences clones D12 and D61 belong to the evolutionary unit ‘C’ and E1 to ‘D’ as denoted by Papakostas et al. (2016). Stock cultures were maintained at room temperature under continuous light conditions and fed daily with the nutrient replete green alga *Chlamydomonas reinhardtii* (1000 μmol C L^-1^). Every 3 days the rotifers were transferred to new containers with fresh medium.

All experiments were based on a comparison between three different food quality treatments: (1) algae cultured in high phosphorus conditions (molar C:P = 112 ± 2.6 SE, further referred to as ‘HP’), (2) algae cultured in low P conditions (molar C:P = 631 ± 14.9 SE, ‘LP’) and (3) algae cultured in low-P media which was then spiked with inorganic phosphate prior to feeding to the rotifers (molar C:P = 113 ± 2.7 SE, LP+P). LP+P algae thus combined high P content with a history of growth under P-limited conditions.

*Chlamydomonas reinhardtii* was cultured in 10 continuous 2L-chemostats at 23 ± 1°C using modified WC (Woods Hole Chu-10) medium (Guillard and Lorenzen, 1972) at a dilution rate of 0.33/day (Appendix S1, Supplementary Figure S1). Five replicate chemostats with HP algae were cultured in media with 65 μmol L^-1^ P under ≈40 μmol quanta m^-2^ s^-1^ of continuous light. Five replicate chemostats with LP algae were cultured in media with 15 μmol L^-1^ P under ≈120 μmol quanta m^-2^ s^-1^ of continuous light. All chemostats were at steady state for at least 1 month prior to the experiments.

The algae for the HP and LP treatments were harvested daily from the chemostats, centrifuged (2500 rpm for 10 min) and resuspended in nutrient free WC medium. To create the LP+P treatment, inorganic phosphate (K_2_HPO_4_, 0.05 mol L^-1^) was added to centrifuged and resuspended LP algae 90 min before being fed to experimental rotifer cultures. The amount of added P was based on the algal C content estimated from cell counts (Multisizer 3^TM^ Coulter Counter, Beckman Coulter). For all three treatments, algae were kept in the dark for 90 min between their harvest and the feeding of the rotifers.

### Population Level Growth Rate Experiment

Population growth rate in each food quality treatment (HP, LP, and LP+P) was estimated for all clones at *ad libitum* food concentrations. Each clone by food treatment had five resource replicates (45 experimental units, i.e., 3 clones × 3 food quality treatments × 5 chemostat replicates). Experimental units were initiated by randomly selecting 10 juvenile rotifers from a stock culture and transferring them into a 16 mL well filled with 8 mL of WC medium containing 1000 μmol C L^-1^ algae. Over the course of 22 days, wells were checked every 24 h and the number of females counted. After counting, each unit was reinitiated by transferring ten haphazardly selected individuals to a new plate with fresh medium. Only juveniles, females without eggs or females with parthenogenetic eggs were transferred, males or females with sexual eggs were not transferred. Plates were incubated at 23°C under continuous darkness.

### Life Table Experiments

Using a life table experiment, we studied the effect of the three food quality treatments on rotifer life history. The design of the life table experiment consisted of a total of 225 experimental units, i.e., 3 resource qualities × 5 food chemostat replicates × 15 individuals. For reasons of feasibility and because all clones showed similar response patterns to the food quality treatments in the growth rate experiment, we only used one clone, D12.

To initiate the experiment, we used cultures as described for the growth rate experiment as a starting point. For each experimental unit in the life table design we isolated at least 10 females with parthenogenetic eggs from these cultures and transferred them to a new well with the corresponding food treatment. These wells were checked hourly for newly hatched neonates over the course of 8 h. Once observed, a neonate was individually transferred into a 3 mL well with 1 ml of algal suspension (1000 μmol C L^-1^) of the same food quality and incubated at 23 ± 1°C in the dark at random locations in an incubator.

After the initial 8 h of their incubation, animals in all experimental units were checked every 2 h until the conclusion of the experiment. At each time point we recorded the number of eggs, the number of neonates produced during the latest interval (which were then removed), and survival. If an individual produced male eggs they were no longer monitored. In the HP and LP+P treatments individuals were monitored until the production of a fourth neonate. As development was much slower in the LP treatment these individuals were instead monitored for the first 62 h.

To obtain estimates on adult body and egg size at first reproduction, we conducted an additional but shortened version of a life table experiment. This experiment had the same design as the full life history experiment, except that only five individuals were used per resource replicate (75 experimental units, i.e., 3 resource qualities × 5 food chemostat replicates × 5 individuals). Neonates were collected in the same manner as in the life history experiment and checked hourly after 8 h. Gravid individuals were preserved in 4% formalin 2 h after the production of their first egg. Body and egg volume were measured manually under a microscope.

### Algae and Rotifer Stoichiometry

Molar C:P ratios of phytoplankton in the food quality treatments were measured at day 1, 6, 11, 16, and 21 of the growth rate experiment. For the life table experiment, the algal C:P ratios were measured just before and after the experiment. Rotifer density was too low in the growth rate experiment to collect enough animals for elemental analysis. For this reason, we scaled up culture conditions of the growth rate experiment to 200 mL batch cultures. The design of this experiment consisted of 30 units, i.e., 2 clones (D12 and D61) × 3 food quality treatments × 5 food replicates. Flasks with 1000 μmol C L^-1^ of algae were initially seeded with rotifers at a density of 15 individuals mL^-1^. Every other day rotifer density was estimated and a volume representing 3000 rotifers was transferred to a new flask, this volume was then reduced to 20 and 180 mL of fresh media was then added to the vessel. This method allowed rotifers to be cultured in a state of constant exponential growth with *ad libitum* food, similar to the cultures in the growth rate experiment. Prior to elemental analysis rotifer individuals with one egg were isolated in nutrient free WC medium for 1 h to allow emptying of the guts. C and N contents were determined using a FLASH 2000 organic element analyzer (Interscience B.V., Breda, Netherlands), while P content was determined by a QuAAtro segmented flow autoanalyzer (Beun de Ronde, Abcoude, Netherlands). Each of these analyses was based on a sample of 150 individuals. During this experiment we also measured molar C:P ratios of phytoplankton in the food quality treatments at two occasions.

### Data Analysis

Exponential population growth rate was repeatedly calculated for each unit of the population level experiment as $R=\frac{{\mathrm{InN}}_{\text{t}}-{\mathrm{InN}}_{0}}{t}$, where *N*_0_ and *N*_t_ represent the population size at the start and end of each 24-h period. Growth rate for each unit was calculated as the mean growth rate for the last 16 days of the experiment (i.e., the period during which growth rates had stabilized).

Life table data was used to calculate mortality rate of focal individuals and of eggs, age at first egg production, egg development time, and egg production rate. Egg production rate was calculated as the total number of eggs produced per hour during a time interval encompassing at least two egg production events per individual. Finally, for each replicate we calculated the instantaneous population growth rate *r* using the Euler–Lotka equation $1={\mathrm{\sum l}}_{x}*m_{x}*e^{(-r*x_{)}}$ (Stearns, 1992), where *l*_x_ represents the fraction of individuals surviving from birth to age class *x*, and *m*_x_ is the fraction of offspring in age class *x*.

Body volume at first reproduction was calculated as *V_b_* = π ∗ *L_b_* ∗ (*W_b_*/2)^2^, where *L*_b_ and *W*_b_ are body length and width at first reproduction, respectively. The volume of parthenogenetic eggs was calculated with the geometric formula for an ellipsoid: $V_{e}=(\frac{4}{3})*\pi *(L_{e}/2)*{\left(W_{e}/2\right)}^{2}$, where *L*_e_ and *W*_e_ represent egg length and egg width (Appendix S2, Supplementary Figure S2). Somatic growth was estimated as the difference between the body volume of an individual at first reproduction and egg volume of the first egg for the same individual divided by the amount of time to mature from a juvenile to first egg production.

In all experiments, phytoplankton chemostats represented the true level of replication. For population growth rate, intrinsic rate of population increase *r*, phytoplankton and rotifer C:P we obtained one value for each independent replicate. Therefore, we analyzed the effect of food quality on *r* and phytoplankton C:P with one-way ANOVA whereas we evaluated the effect of food quality and its interaction with ‘clone’ on population growth rate and rotifer C:P with a two-way ANOVA. Whereas clone should in fact represent a random factor we still specified it as a fixed factor because it only comprises three levels. In contrast, for all other life history variables we collected data from multiple individuals per chemostat replicate. We accounted for the intrinsic dependency of these data using general linear mixed models. In these models, food chemostat replicates were specified as random factor and food quality as fixed factor. For all life history variables the significance of food quality was evaluated with a likelihood ratio test comparing the full model with the corresponding intercept model. All ANOVA and linear mixed models were studied in more detail with Tukey *post hoc* comparisons to assess the significance of differences among factor levels. All statistical analyses were performed in R software environment 3.3.1 (R Core Team, 2016). Mixed effects analyses were performed with the lme4-package (Bates et al., 2014) in R (R Core Team, 2016).

## Results

### Growth Rate Experiment

**FIGURE 2:**
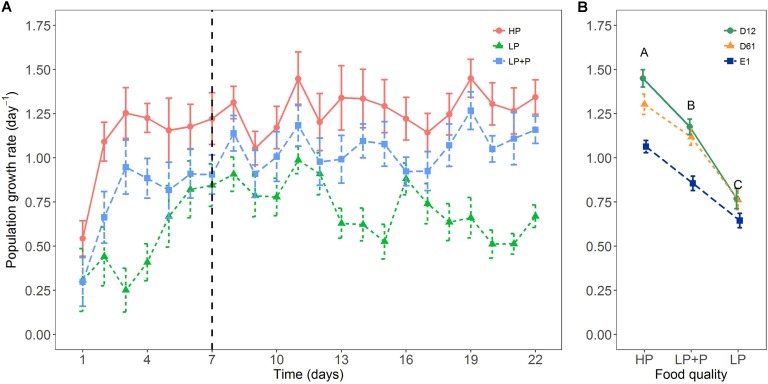
Response of rotifer population growth rates to the three food quality treatments in the growth rate experiment. **(A)** Mean growth rate of the different food treatments for each day over the course of the experiment and **(B)** mean population growth rate (Days 7–22) of the three clone lines. Circles represent clone D12, triangles clone D61 and squares clone E1. HP: algal food cultured in P-replete conditions; LP: algal food cultured in P-depleted conditions; LP+P: LP algae spiked with inorganic phosphate just before feeding. Different letters indicate significant differences among food treatment levels as tested with a Tukey *post hoc* comparison across clones. Symbols and error bars represent the mean ± 2 standard error, respectively.

Food quality had a strong effect on rotifer population growth rates (**Figure 2A**). A two-way ANOVA detected a significant interaction between food quality and clone identity for mean population growth rate (**Table 1**): growth rate differences among clones were clearly expressed in the HP and LP+P treatments, however, such differences proved relatively small in the LP treatment (**Figure 2B**). Yet, all clones showed a very similar response pattern to the food quality treatments: the HP treatment had the highest mean population growth rate, while the LP+P treatment was intermediate to the HP and LP treatments.

**Table 1:** Summary of ANOVA results for population growth rate and algal and rotifer C:P ratios.

	SS	MS	df	*F*-value	*p*
**Population-level growth rate experiment**					
Population growth rate			
Food	2.27	1.14	2	478.4	<0.001
Clone	0.62	0.31	2	144.1	<0.001
Food ^∗^ Clone	0.14	0.04	4	11.9	<0.001
**Life table experiments**					
Intrinsic growth rate *r*			
Food	0.04	0.02	2	21.9	<0.001
**Algae and rotifer stoichiometry**			
Algal C:P ratio			
Food	7.23 × 10^5^	3.61 × 10^5^	2	119.8	<0.001
Rotifer C:P ratio					
Food	3.37 × 10^4^	1.69 × 10^4^	2	179.6	<0.001
Clone	80.0	80.0	1	0.9	0.365
Food ^∗^ Clone	1609	804	2	8.6	0.002

*Note that an additional factor ‘clone’ was incorporated in the analyses of population growth rate and rotifer C:P. SS, sum of squares; MS, mean square; df, degrees of freedom.*

### Life Table Experiments

**FIGURE 3:**
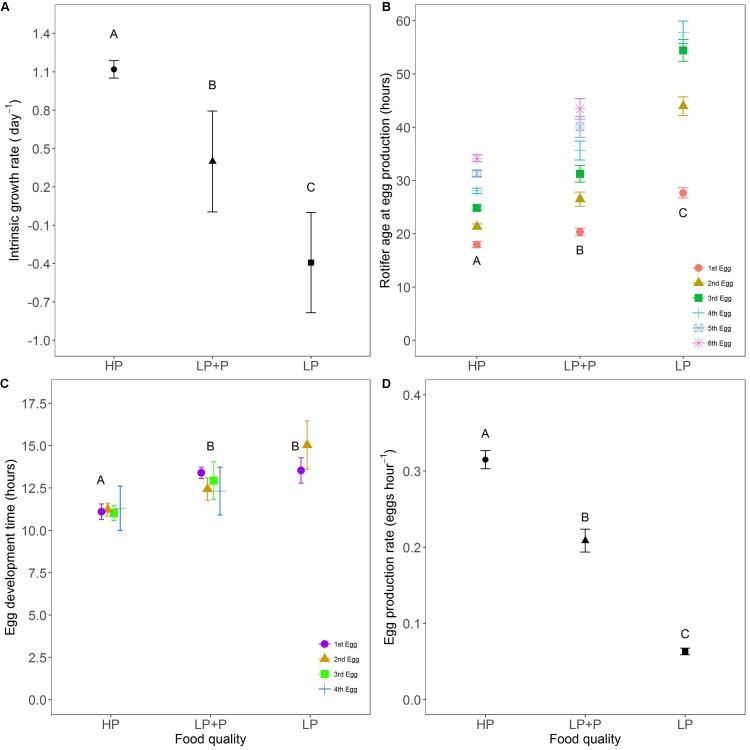
Life history traits in response to food quality treatments. **(A)** Intrinsic population growth rate **(B)** age at egg production, **(C)** egg development time, and **(D)** egg production rate. HP: algal food cultured in P-replete conditions; LP: algal food cultured in P-depleted conditions; LP+P: LP algae spiked with inorganic phosphate just before feeding. Different letters indicate significant differences among food treatment levels as tested with a Tukey *post hoc* comparison. Letters in **(B,C)** only represent analysis results for the first eggs produced. Symbols and error bars represent the mean ± 2 standard error, respectively.

The intrinsic rate of population increase *r* was significantly different between all treatment combinations (**Figure 3A** and **Table 1**). *r* was highest in the HP, lowest in the LP and intermediate in the LP+P treatment (*post hoc* test: HP-LP, *p* < 0.001, HP-LP+P, *p* = 0.021, LP+P-LP, *p* = 0.012). *r*-values were positive in the HP and LP+P treatments but negative in the LP treatment.

The mortality rate of experimental individuals was 8.0% in the LP, 1.4% in the LP+P and 0% in the HP treatment. Larger differences were observed in egg mortality where 23.1% of rotifer eggs died before hatching in the LP treatment, in contrast to the HP and LP+P treatments where no eggs died.

The age at first egg production was lowest in the HP and highest in the LP treatment [**Figure 3B** and **Table 2**, χ^2^(2) = 148.07, *p* < 0.001]. Although values for this variable were higher in the LP+P treatment than in the HP treatment, they approached more those of the HP than of the LP treatment (**Figure 3B** and **Table 3**). A similar pattern was found for the ages at which subsequent eggs were produced. The development time of first egg was similar in the LP and LP+P treatments and longer than in the HP treatment [**Figure 3C**; χ^2^(2) = 24.384, *p* < 0.001; **Tables 2**, **3**]. The development time of subsequent eggs differed significantly among all treatments (**Figure 3C**). Egg production rate was highest in the HP and lowest in the LP [χ^2^(2) = 338.67, *p* < 0.001; **Table 2**]. Egg production rate in the LP+P treatment was intermediate but approached more that of the HP treatment (**Figure 3D** and **Table 3**).

**Table 2:** Summary of mixed model analyses for life table results.

Fixed Effect	SS	MS	df	*F*-value
**Life table experiment**				
Age at first egg production				
Food quality	2372	1186	2	166.7
Development time of first egg				
Food quality	90.2	45.1	2	14.2
Egg production rate				
Food quality	1.68	0.84	2	604.5
Body size at first egg production				
Food quality	3.40	1.70	2	7.8
Size of first egg				
Food quality	0.18	0.09	2	7.8
Somatic growth rate				
Food quality	0.07	0.03	2	59.6

*Food quality was specified as fixed effect in the models. SS, sum of squares; MS, mean square; df, degrees of freedom. P-values were obtained through application of the ratio likelihood test and are reported in the text of the section “Results.”*

**Table 3:** Overview table with estimates of the relative impact of direct and indirect effects of P limitation on the investigated traits of *B. calyciflorus*.

Traits	Effect source	Relative differences	*p*
**Population growth rate**			
(LP+P)-HP	Indirect	-17.5%	<0.001
LP-(LP+P)	Direct	-25.0%	<0.001
**Somatic growth rate**			
(LP+P)-HP	Indirect	-18.6%	0.001
LP-(LP+P)	Direct	-27.4%	<0.001
**Age at first egg production**			
(LP+P)-HP	Indirect	19.1%	<0.001
LP-(LP+P)	Direct	45.0%	<0.001
**Development time of first egg**			
(LP+P)-HP	Indirect	20.7%	<0.001
LP-(LP+P)	Direct	1.2%	0.940
**Egg production rate**			
(LP+P)-HP	Indirect	-33.8%	<0.001
LP-(LP+P)	Direct	-46.1%	<0.001
**Egg mortality**			
(LP+P)-HP	Indirect	0.0%	1
LP-(LP+P)	Direct	-23.1%	0.007
**Body size at first egg production**			
(LP+P)-HP	Indirect	-1.9%	0.889
LP-(LP+P)	Direct	-12.3%	0.007
**Size of first egg**			
(LP+P)-HP	Indirect	36.2%	0.005
LP-(LP+P)	Direct	2.9%	0.956
**Rotifer C:P ratio**			
(LP+P)-HP	Indirect	5.3%	0.67
LP-(LP+P)	Direct	90.6%	<0.001
**Rotifer C content**			
(LP+P)-HP	Indirect	14.6%	0.01
LP-(LP+P)	Direct	-39.8%	<0.001
**Rotifer P content**			
(LP+P)-HP	Indirect	8.3%	0.14
LP-(LP+P)	Direct	-70.3%	<0.001

*The relative impact of direct effects was calculated as (μ_LP-_μ_LP+P_)/μ_HP_.100, whereas the relative impact of indirect effects was calculated as (μ_LP+P-_μ_HP_)/μ_HP_.100, where μ refers to the mean value across replicates of a food quality treatment. Negative signs indicate reductions in trait values. P-values were obtained through Tukey post hoc comparisons.*

**FIGURE 4:**
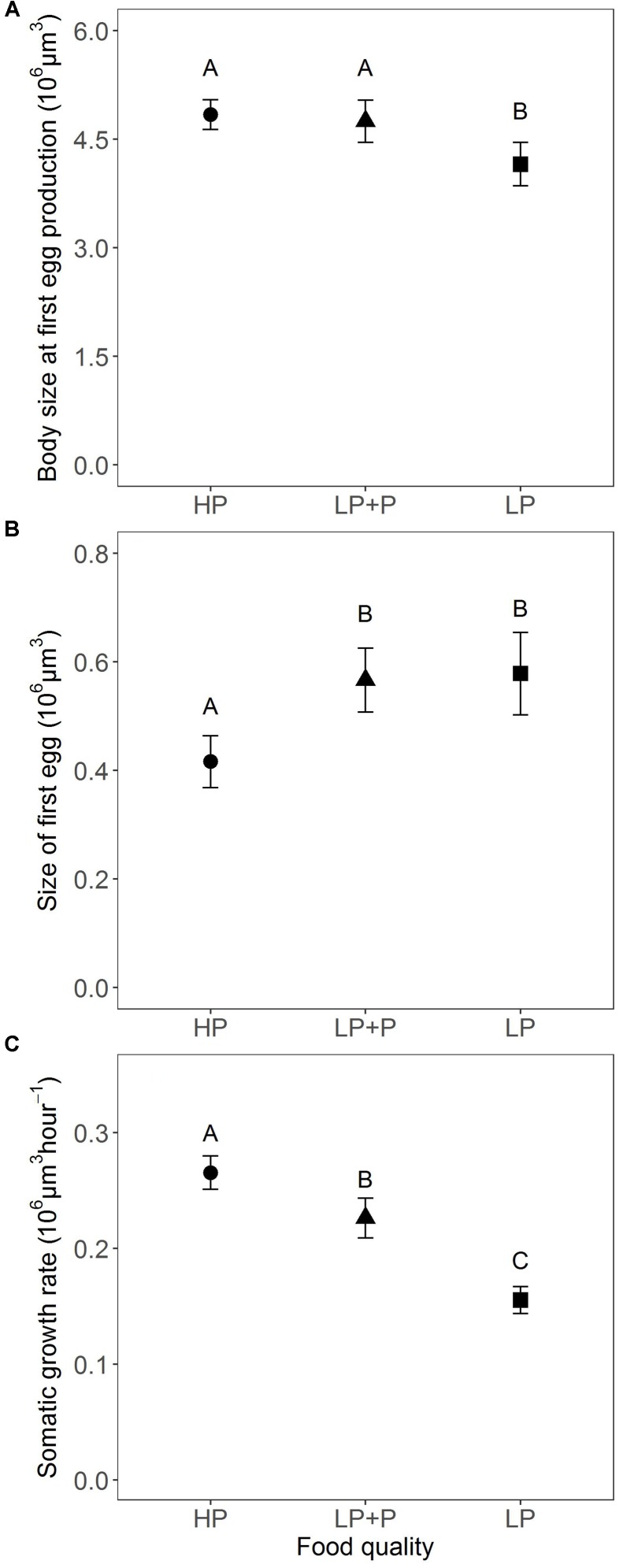
Size-related traits in response to food quality treatments. **(A)** Estimated body size at first egg production, **(B)** estimated size of first egg, and **(C)** estimated somatic growth rate. HP: algal food cultured in P-replete conditions; LP: algal food cultured in P-depleted conditions; LP+P: LP algae spiked with inorganic phosphate just before feeding. Different letters indicate significant differences among food treatment levels as tested with a Tukey *post hoc* comparison. Symbols and error bars represent the mean ± 2 standard error, respectively.

Body size at first egg production in the HP did not differ significantly from the LP+P treatment (**Figure 4A**). However, in both treatments body size was significantly larger than in the LP treatment [χ^2^(2) = 12.983, *p* < 0.002; **Tables 2**, **3**]. In contrast, the size of first egg was not significantly different between the LP+P and LP treatments (**Figure 4B** and **Table 3**), but in both treatments it was significantly larger than in the HP treatment [χ^2^(2) = 12.931, *p* < 0.002; **Table 2**]. Somatic growth rate differed among all three treatments [**Figure 4C**; χ^2^(2) = 51.508, *p* < 0.001]. Somatic growth rate was highest in the HP treatment and intermediate in the LP+P treatment (**Tables 2**, **3**).

### Algal and Rotifer Stoichiometry

**FIGURE 5:**
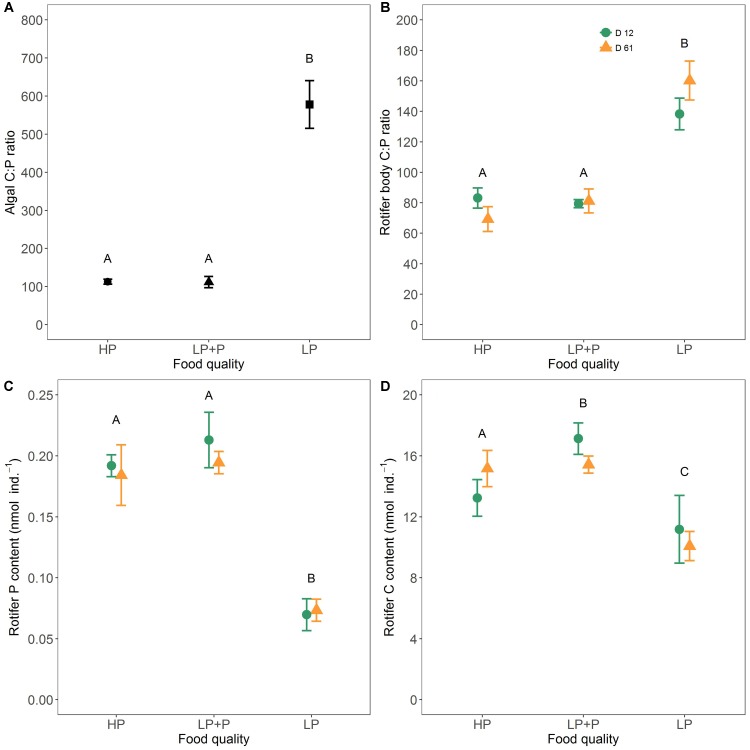
Stoichiometric ratios of algal food and rotifers. **(A)** Averages over time of the molar C:P ratios of the three food quality treatments, **(B)** body C:P ratios of rotifers raised on the three food quality treatments, **(C)** body P content of rotifers raised on the three food treatments, and **(D)** body C content of rotifers raised on the three food treatments. Green circles represent clone D12, and yellow triangles clone D61. Different letters indicate significant differences among food treatment levels as tested with a Tukey *post hoc* comparison across clones. Symbols and error bars represent the mean ± 2 standard error, respectively.

Throughout the experiment the C:P ratio of the LP algae was much higher than in the other two treatments (**Figure 5A** and **Table 1**). No significant difference in the C:P ratio was observed between the HP and LP+P treatment.

A significant interaction between food quality treatment and clone was observed for rotifer body C:P ratio as well as body P and C content (**Figures 5B–D** and **Table 1**). However, both clones showed a very similar response to food quality and the majority of the variation was explained by the food quality treatment (**Table 1**). The body C:P of rotifers from the LP treatment was significantly higher than of rotifers from the HP and LP+P treatments. No significant difference in the C:P ratio was observed between the HP and LP+P treatment. These patterns were driven by variation in total body P (**Table 3**). Animals in the LP treatment contained less C than animals in the HP and LP+P treatments (**Table 3**). Nevertheless, their C:P values were higher due to a proportionally very low P content (**Figures 5C,D** and **Table 3**).

## Discussion

In line with previous work (Rothhaupt, 1995; DeMott, 1998; Boersma, 2000; Becker and Boersma, 2003), our P-supplementation study shows that P-limitation of primary producers negatively affects zooplankton consumers not only directly through a reduced availability of P, but also indirectly via non-stoichiometric, qualitative effects. Indeed, general performance measures of rotifers, such as somatic and population growth rates proved to be affected almost as strongly by indirect as by direct effects (**Table 3**). Novel to our study is that we were able to evaluate the relative importance of these direct and indirect effects on multiple life history traits simultaneously. Intriguingly, the response of these traits proved to differ very strongly. Some traits such as size and age at first reproduction and egg mortality were largely affected by the direct effects of P-shortage, whereas other traits (e.g., egg size and first egg development time) seemed only affected by indirect effects of P-limitation. The P content and C:P ratio of rotifers fed P-supplemented LP algae (LP+P) was equally high as in rotifers fed HP algae. This indicates that the observed reduction of rotifer performance in the LP+P compared to the HP treatment cannot be explained by a lower accessibility of P in LP+P food.

Animals provided with P-limited algae had lower somatic growth rate, older age of maturity, lower egg production rate, longer egg development time and higher egg mortality compared to animals grown with P-rich algae. These responses are largely in line with other studies reporting the effects of P-limitation on zooplankton life history, although most of such work has been done on *Daphnia* (Urabe and Sterner, 2001; Færøvig and Hessen, 2003; Lukas et al., 2013). To our knowledge, there are only two studies reporting on the impact of P-limitation on rotifer life history. When feeding *B. calyciflorus* P-limited algae, Jensen and Verschoor (2004) observed a lower somatic growth rate, an older age at first egg production and a shorter reproductive period compared to animals fed P-replete algae although egg mortality and total life span remained unaffected. Conversely, in a study of the rotifer *Keratella cochlearis*, Ramos-Rodríguez and Conde-Porcuna (2003) observed a lower offspring production, a higher age at maturity, and a lower life span in animals fed with P-replete compared to P-limited *Cryptomonas* algae. However, in this experiment the C:P of the nutrient sufficient *Cryptomonas* was higher than that of the P-limited *Cryptomonas.*

In our study, the enhancement of growth performance following supplementation of P-limited algae with inorganic P supports the idea that consumer productivity is strongly impacted by the quantitative lack of P and the associated stoichiometric imbalance. However, our results also indicate that such direct effects of P-limitation cannot fully explain the decreased performance of rotifers under P-limited food conditions. The C:P ratio of algae in the LP+P treatment was equal to that of the HP algae. Similarly, the body P content and the C:P ratio of adult rotifers fed LP+P food was similar to that of animals fed with HP food, and both were substantially different from rotifers in the LP treatment. We therefore conclude that it is unlikely that morphological changes induced by a history of P-limitation or that the form of P-storage in LP+P algae has reduced accessibility of P to the consumers. Nevertheless, population growth rate remained considerably lower than in rotifers fed HP algae. This result suggests that P-limitation induced non-stoichiometric qualitative changes in phytoplankton which negatively affected its suitability as food for zooplankton.

Our results are in line with a number of other P-supplementation studies (Rothhaupt, 1995; DeMott, 1998; Boersma, 2000; Becker and Boersma, 2003) which suggested important indirect effects of food P-limitation on zooplankton consumer performance. Furthermore, through our life table data, we are able to assess the relative importance of direct stoichiometric and indirect non-stoichiometric effects of algal P-limitation on multiple fitness components, simultaneously. Most life history traits seemed to respond to P-addition, but still bore a clear signature of indirect effects of P-limitation. Similar to the population growth rates measured in the population-level culture experiment, somatic growth rate and intrinsic rate of population increase reached values in the LP+P treatment that were intermediate to that in the LP and HP treatments. Similarly, egg production rate and age at first egg production in the LP+P treatment were also intermediate to LP and HP although they appeared to be more strongly influenced by P addition because their values approached more those of the HP than the LP treatment.

However, other traits deviated strongly from such pattern. Both size at first reproduction and egg mortality in the LP+P treatment equaled that of the HP treatment, suggesting these traits are exclusively impacted by the direct effects of P-limitation. Conversely, size and development time of the first egg showed no response to P-addition and appeared to be entirely controlled by indirect effects of P-limitation. Our results therefore clearly demonstrate a differential sensitivity of different fitness components to indirect and direct effects of P-limitation in the food resource. Likely this is reflective of the fact that both stoichiometric (Urabe and Sterner, 2001; Villar-Argaiz et al., 2002; Becker and Boersma, 2003; Færøvig and Hessen, 2003) and biochemical requirements (Martin-Creuzburg and Von Elert, 2004; Wacker and Martin-Creuzburg, 2007) vary among the different predominant physiological processes that characterize ontogenetic stages of the consumers. For example, fast somatic growth of juvenile stages is known to be highly dependent on the availability of P (cf. ‘growth rate hypothesis,’ Elser et al., 2003). In contrast, egg development may be more dependent on the availability of specific biochemical substances. For example, *Daphnia* eggs have been shown to contain disproportional amounts of fatty acids compared to somatic tissue (Wacker and Martin-Creuzburg, 2007), especially polyunsaturated fatty acids (PUFA’s) such as eicosapentaenoic acid (EPA). Wacker and Martin-Creuzburg (2007) demonstrated that poor biochemical quality of food reduced the amount of these essential fatty acids in *Daphnia* eggs, and suggested an important role of biochemical compounds for egg development. Possibly, the slower development rate of eggs in the LP+P and LP treatments may have been the result of lower biochemical quality. We can only speculate about the mechanisms that may underlie our observation of larger eggs in the LP and LP+P treatments compared to the HP treatment. Larger eggs often reflect increased allocation of carbon resources of the mother to its progeny (Gliwicz and Guisande, 1992; Kirk, 1997). It is possible that mother animals in the LP treatment discarded excess C into their eggs (Urabe and Sterner, 2001). Rotifers of clone D12 contained more C in the LP+P treatment than in the HP treatment, despite equal C-availability and C:P ratio of these food treatments. Possibly, the larger egg size observed in the LP+P treatment also reflected a C allocation strategy of adults toward their eggs similar as in the LP treatment.

Morphological changes in phytoplankton have also been suggested to be the cause of reduced consumer performance under conditions of P-limitation. Algae have been reported to respond to nutrient limitation with an increase in cell size (van Donk and Hessen, 1995) and increased thickness of their cell wall (van Donk and Hessen, 1993; van Donk et al., 1997). In filter feeders like *Daphnia*, these morphological changes improve viable gut passage and explain reduced clearance and population growth rates of these grazers when fed P-limited algae (Lürling and van Donk, 1997; van Donk et al., 1997). However, although cell size increased in response to P-limitation in our experiment, they remained well within the limits of the food particle size range ingestible for *B. calyciflorus* (Rothhaupt, 1990). Additionally, in contrast to *Daphnia*, rotifers crush ingested food with a specialized stomach (mastax; Gilbert and Starkweather, 1977), hence, it is doubtful that cell wall thickening would allow gut passage of intact cells. Rothhaupt (1995) observed no reduction in grazing rates of *B. rubens* on P-limited algae, whereas P-limitation has also been found to result in increased clearance rates (Suzuki-Ohno et al., 2012). Finally, in our experiment, rotifer body C and P content did not decrease in the LP+P compared to the HP treatment, suggesting no reduction in C and P ingestion and assimilation efficiencies.

Our study highlights that the performance of consumers provided with a phosphorus limited resource is not exclusively affected by the quantitative reduction of available P and the corresponding stoichiometric mismatch with their elemental requirements. Consumer performance was also impacted by the qualitative deterioration of the food as a result of the resource growth environment that acted independently of elemental content or stoichiometric ratios of the final food resource. In our study, such indirect qualitative effects proved to contribute strongly to the observed reductions in consumer population growth under P-limited conditions. Importantly, the magnitude of the impact of these indirect effects seemed to differ between different key fitness components of consumers. Given the strong link between life history and population demography, this suggests that such effects may also have an important impact on the structure and dynamics of consumer populations. Furthermore, the relatively large impact of the indirect effects of P-limitation in our results highlight their potential importance in determining the strength of producer-consumer bottom-up control and the efficiency of energy transfer between trophic levels. A better knowledge of the consequences of non-stoichiometric food quality effects of P-limitation on consumer populations may therefore be crucial for a better understanding of the true nature of P-limitation effects in natural communities.

## Author Contributions

LZ and SD developed the idea and designed the experiments. LZ carried out the growth rate and batch culture experiments. LZ, KL, WZ, and SD conducted the life table experiment. Data analysis was mainly performed by LZ and KL. LZ, KL, and SD wrote the manuscript.

## Conflict of Interest Statement

The authors declare that the research was conducted in the absence of any commercial or financial relationships that could be construed as a potential conflict of interest.
