# Supplementary material for: Direct and Indirect Effects of Resource P-Limitation Differentially Impact Population Growth, Life History and Body Elemental Composition of a Zooplankton Consumer
Source: Front Microbiol. 2018 Feb 9;9:172. doi: 10.3389/fmicb.2018.00172 (PMC5811457; doi:10.3389/fmicb.2018.00172)
Supplement: Supplementary file 1 [file Image_1.pdf]

Supplementary Material

Direct and indirect effects of resource P-limitation differentially impact population growth, life history and body elemental composition of a zooplankton consumer

Libin Zhou, Kimberley Lemmen, Wei Zhang, Steven A.J. Declerck

*** Correspondence: Libin Zhou: L.Zhou@nioo.knaw.nl**

Appendix S1 Additional methodological information of phytoplankton chemostat set-up

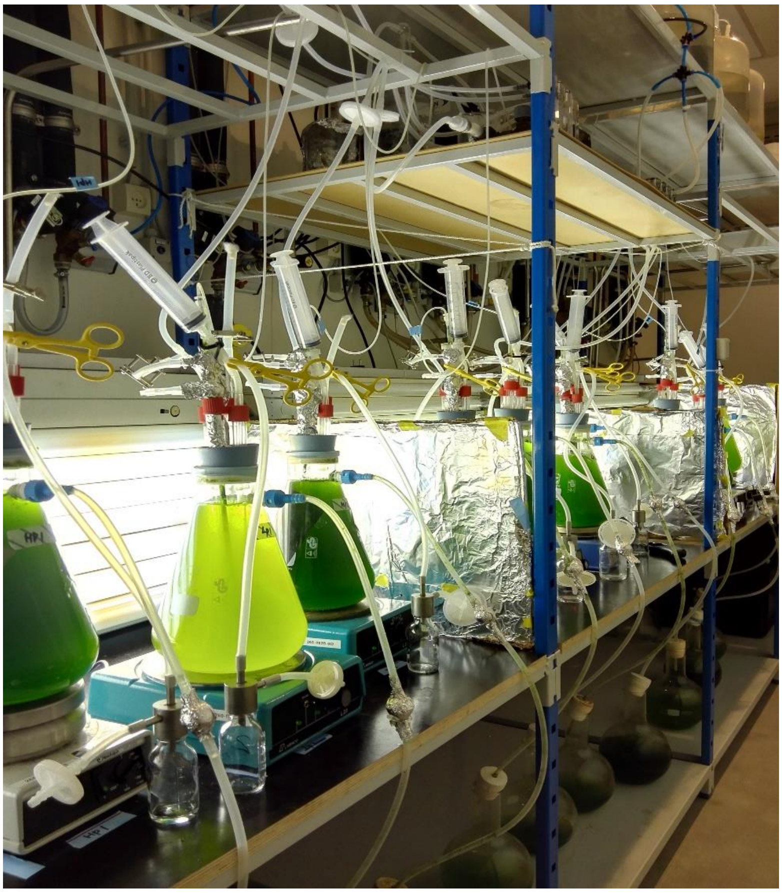

Figure S1. Photograph of the phytoplankton chemostat system under two different nutrient conditions。

Appendix S2 Measurement of body size and egg size of rotifer *B. calyciflorus* (only measure animal with the front position)

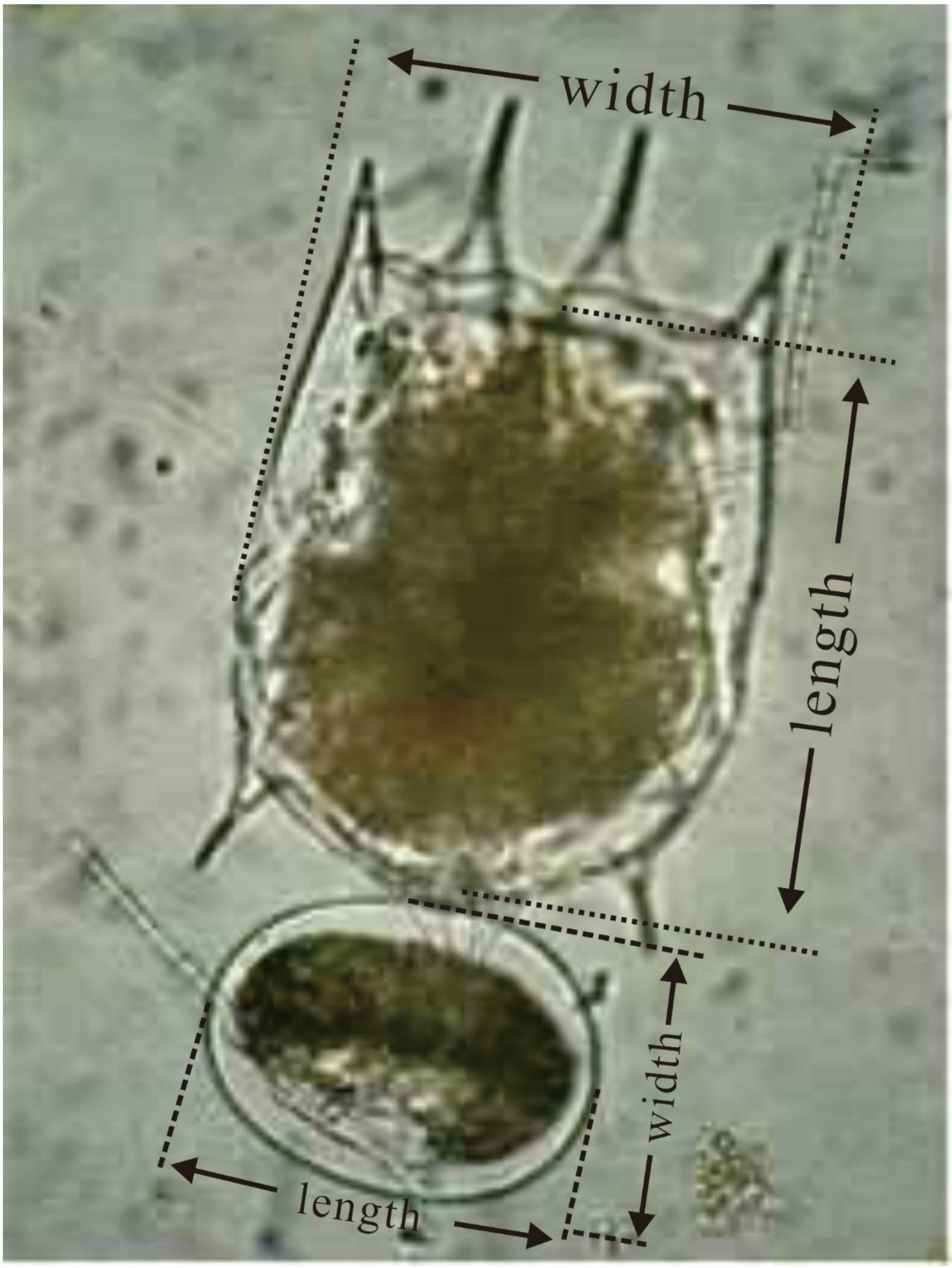

Figure S2. Photograph for the measurement of rotifer body and egg size
